# 

**Published:** 1889-04

**Authors:** 


### American Medical Association.

The fortieth annual meeting of this body will be held in Newport, R. I., on the 25th, 26th, 27th, and 28th of June, 1889.

It will be well for all interested to remember, that the time of this meeting is rapidly approaching, and those who are to take part in its work should bear this fact in mind. And especially would we impress this on the attention of the members of the Dental Section, or those who are to become members thereof.

All members, and others who contemplate contributing papers to the sections, should communicate with the officers of the section in which their papers will be read as soon as possible. The Secretary of the Dental Section reports that he has already several important papers promised, and from parties who will not fail to fulfill their promise. A much greater interest is now being taken in this section than ever before; and we trust it will be largely attended this year.

Every paper presented and read is considered the exclusive property of the association. All papers should be so prepared, that they may be put into the hands of the Permanent Secretary at once, to be transmitted to the Committee of Publication. Dr. C. A. Brackett, of Newport, R. I., is a member of the local committee and doubtless will be pleased to give information to any member of the dental section.

The officers of the Section on Dental and Oral Surgery are Dr. F. H. Rehwinkel, of Chillicothe, Ohio, President; and Dr. E. S. Talbot, of Chicago, Secretary. The next number of the REGISTER will contain a list of the papers to be read in that section.

THE American Association for the advancement of Science will meet in Toronto, August 27 to September 3.

### CONTENTS.

COMMUNICATIONS.

A MONTHLY MAGAZINE DEVOTED TO THE INTERESTS OF THE

**DENTAL PROFESSION.**

*Terms Reduced to* $2.00 *Per Year. Single Copies,* 25 *Cents.*

**EDITED BY PROF. J. TAFT, D.D.S.**

—AND—

**Published by SAM'L A. CROCKER & CO., Ohio Dental and Surgical Depot.**

The volume commences in January and closes in December.

The REGISTER being issued monthly is always fresh, therefore Indispensable to the practitioner who desires to keep posted up with the new inventions and improvements which are daily developed. Neither expense nor effort will be spared to give value and variety to its contents. Articles will be illustrated with well executed engravings whenever they will add to the interest or assist to explanation.

Its extensive circulation and frequency of issue make it one of the BEST DENTAL ADVERTISING MEDIUMS in the United States.

All subscriptions must begin with January or July.

Local or special notices, five lines or less, will be inserted for $3 each insertion; over five lines, 50 cts. per line.

All advertising bills payable in advance, or at furthest, after the issue of the first number containing the advertisement. All transient advertisements to be paid for in advance.

☞CONTRIBUTIONS SOLICITED.

Exclusively Editorial Communications should be addressed to the Editor.

The latest number of the REGISTER may be ordered with or without other goods at any time, and all letters should be addressed to

SAM'L A. CROCKER & CO., Ohio Dental and Surgical Depot, Cincinnati, O.

